# Association between community-based services, psychological resilience, and cognitive impairment among older adults in China

**DOI:** 10.1038/s41598-025-03491-w

**Published:** 2025-07-01

**Authors:** Suyan Wang, Xiyu Chen, Haoyu Wang, Qiwei Tang, Cheng Cheng, Yao Wu, Jinrui Hu, Yishan Duan, Xiaobing Xian, Bojiang Chen

**Affiliations:** 1 Frontiers Science Center for Disease-related Molecular Network, West China Hospital, and Key Laboratory of Bio-Resource and Eco-Environment of Ministry of Education, College of Life Sciences, Sichuan University, Chengdu, 610041 Sichuan China; 2 Precision Medicine Center, Precision Medicine Key Laboratory of Sichuan Province, West China Hospital, Sichuan University, Chengdu, 610041 Sichuan China; 3 The First Clinical College, Chongqing Medical University, Chongqing, 400016 China; 4 International Medical College, Chongqing Medical University, Chongqing, 400016 China; 5 The Thirteenth People’s Hospital of Chongqing, Chongqing, 400053 China; 6 Chongqing Geriatrics Hospital, Chongqing, 400053 China

**Keywords:** Community-based services, Psychological resilience, Cognitive impairment, Older adults, Structural equation model, Health care, Public health

## Abstract

Cognitive impairment (CI) has emerged as a critical global public health concern, particularly among older adults. Community-based services (CBSs) and psychological resilience (PR) are potential protective factors, yet their interrelationships remain underexplored. This study examines how CBSs influence CI among older adults and whether PR mediates this association. Based on data from 7,565 individuals aged ≥ 65 in the China Longitudinal Health Longevity Survey (CLHLS), the structural equation modeling was used to test direct and indirect pathways between CBSs, PR, and CI, adjusting for eighteen basic demographic covariates. Mediation effects were validated via the Bootstrap 2000 method. The findings highlight that CBSs are positively associated with both lower CI (*β* = 0.049, *P* < 0.001) and higher PR (*β* = 0.071, *P* < 0.001), while PR associates with CI risk positively (*β* = 0.260, *P* < 0.001). PR mediated 26.87% of the total CBSs-CI association (indirect effect = 0.018, 95%CI: 0.012–0.026). Specific CBSs, including doctors or sending medicine to home (*χ*^2^ = 7.05, *P* = 0.008) and health care education (*χ*^2^ = 7.89, *P* = 0.005), showed a strong protective effect against CI. These results underscore the importance of enhancing CBSs and fostering PR to mitigate CI and promote healthy aging. A collaborative effort between communities and society is essential to empower older adults, strengthen their PR and facilitate active aging.

**Supplementary Information:**

The online version contains supplementary material available at 10.1038/s41598-025-03491-w.

## Introduction

According to the Seventh National Population Census, China has the largest old population in the world, with 264 million people aged 60 and above, accounting for 18.70% of the total population^1^. This poses higher demands on Chinese society to prioritize the health of older adults and develop relevant healthcare strategies for promoting healthy and active aging^2^. As the phenomenon of aging continues to intensify, greater emphasis is being placed on the physical and mental health of older adults, particularly cognitive disorders. Cognitive impairment (CI) is an age-related neurodegenerative disorder characterized primarily by a decline in higher brain functions, including memory, executive function, and language, which can significantly affect individuals’ daily living activities to varying degrees^3^. Previous studies have demonstrated that CI substantially increases the risk of dementia among older adults and has become a major leading cause of disability^4^. Consequently, a better understanding of the key influencing factors and underlying mechanisms of CI among older adults is critical for developing feasible interventions and lowering the disease burden attributable to CI.

In response to mitigate the practical pressures arising from changes in family structures and further facilitate active aging in China, the government has implemented multifaceted measures, among which promoting the development of community-based services (CBSs) constitutes a key component^5,6^. As a meso-level mediator connecting macro-level policies and micro-level individuals, communities provide essential service resources that constitute fundamental support systems, with a unique advantage in spatial accessibility and service continuity. These core services encompass, but are not limited to daily care, medical assistance, psychological support, and legal aid, which collectively are associated with improved care provision and enhanced social inclusion among older adults^7,8^. Previous research has focused on the impact of macro-level social services on CI, with limited investigation having explored this association at the community level^9,10^. Notably, a 2023 study based on a Chinese population revealed that home and CBSs were significantly associated with reduced prevalence of CI among older adults^11^. However, the mechanisms through which CBSs may affect CI remain unclear.

Psychological resilience (PR) is defined as an individual’s ability to maintain psychological stability and achieve adaptive development in the face of adversity^12^. Previous research has demonstrated a close relationship between PR and CI among older adults^13^. A prospective study by Wolf and Cohen et al. revealed that individuals with lower PR scores exhibit more pronounced amyloid-*β*-associated cognitive decline^14,15^. A study published in 2023 based on the Chinese Longitudinal Healthy Longevity Survey (CLHLS) database further confirmed that higher PR scores are significantly associated with a reduced risk of CI, providing epidemiological evidence to support the association between PR and CI^16^. Regarding the relationship between CBSs and PR, Ozbay et al. pointed out that individuals can fully utilize social support to counter external stressors and thereby achieve better social psychological functions in 2007^17^. The support provided by CBSs from the community may have certain similarities with social support. Additionally, the Psychological Resilience Framework Model proposed by psychologist Kumpfer in 1999, based on the ecological system theory, emphasizes that PR results from the dynamic interaction between the individual and the environment^18^. It also holds that protective resources provided by the external environment are the foundation for enhancing PR. Considering that CBSs include services such as home care, psychological counseling, and health lectures for the elderly, they essentially provide social support from multiple perspectives such as instrumental, emotional, and supportive, which may reduce the stress load of older adults and enhance their social support network, thereby improving their PR^19,20^.

Based on the above, this study innovatively proposes the hypothesis that CBSs may further reduce the occurrence of CI by improving the PR among older adults. The ABC emotion theory proposed by American psychologist Ellis in 1957 provides a certain theoretical basis for the construction of our hypothesis. It emphasizes the dynamic interaction system of “Activating Event (A) - Belief (B) - Consequence (C)”, suggesting that by intervening in external supportive events (A), individual cognitive beliefs (B) can be reshaped, thereby improving adverse outcomes (C)^21,22^. Considering that CBSs are an external intervention event, community staff may provide services or support to older adults through this approach, changing the objective environment that affects their cognitive function, which is in line with the characteristics of activating events. Older adults’ evaluation of CBSs forms their beliefs, which are directly related to their ability to cope with different environments, which is also called PR. CI, as an outcome that can be regulated by PR, is consistent with C. This suggests that PR, as an internal belief closely related to cognition among older adults, may play a mediating role between CBSs and CI. That is, CBSs may enhance the positive internal beliefs, promote the improvement of their PR, and ultimately reduce the possibility of CI among older adults.

In summary, this study utilizes cross-sectional data from the CLHLS 2018 to investigate the relationships among CBSs, PR, and CI among older adults in China. Structural equation modeling (SEM) is employed to analyze these relationships, and the Bootstrap method is used to test the mediating effect of PR. Compared to traditional statistical methods such as logistic regression, SEM offers superior model fit and accounts for measurement errors, thereby enhancing the precision of the model. This study innovatively proposed the mediating effect of PR between CBSs and CI. It was the first time that Ellis’s ABC emotion theory was extended and applied to the research on community services. We believe that the findings will provide a solid theoretical foundation for constructing a multi-level (family-community-society) psychological intervention system, supporting the transition of healthy aging strategies from a medical-dependent model to a community-preventive model. Based on the theoretical framework outlined above, we propose the following research hypotheses:

### Hypothesis 1

CBSs have a positive association with reducing CI.

### Hypothesis 2

PR has a positive association with reducing CI among older adults.

### Hypothesis 3

CBSs have a positive association with enhancing PR.

### Hypothesis 4

PR plays a significant mediating role in the relationship between CBSs and CI.

## Materials and methods

### Design

This cross-sectional study examined the associations between CBSs, PR, and CI based on the CLHLS database. Following STROBE statement requirements, we systematically reviewed and refined our manuscript with the EQUATOR reporting checklist to ensure transparent reporting of this observational study.

### Participants

The data used in this study were obtained from the CLHLS, jointly conducted by the Center for Healthy Aging and Development Studies at Peking University and Duke University. As the largest study on health and longevity currently conducted worldwide, the CLHLS included 22 areas in 31 provinces, covering the majority of the Chinese population. It examines the health status, social, behavioral, and biological aspects among older adults^23^. A comprehensive survey was conducted on all participants, covering factors of health such as lifestyle, physical capacity, cognitive function, psychological status, and socioeconomic traits^24^. The research ethics committee at Peking University approved the CLHLS study (IRB00001052-13074) and informed consent was obtained from all participants or their proxy responses. As our research utilized existing data from this database, no new data collection or additional ethical approval was required for this manuscript.

**Fig. 1:**
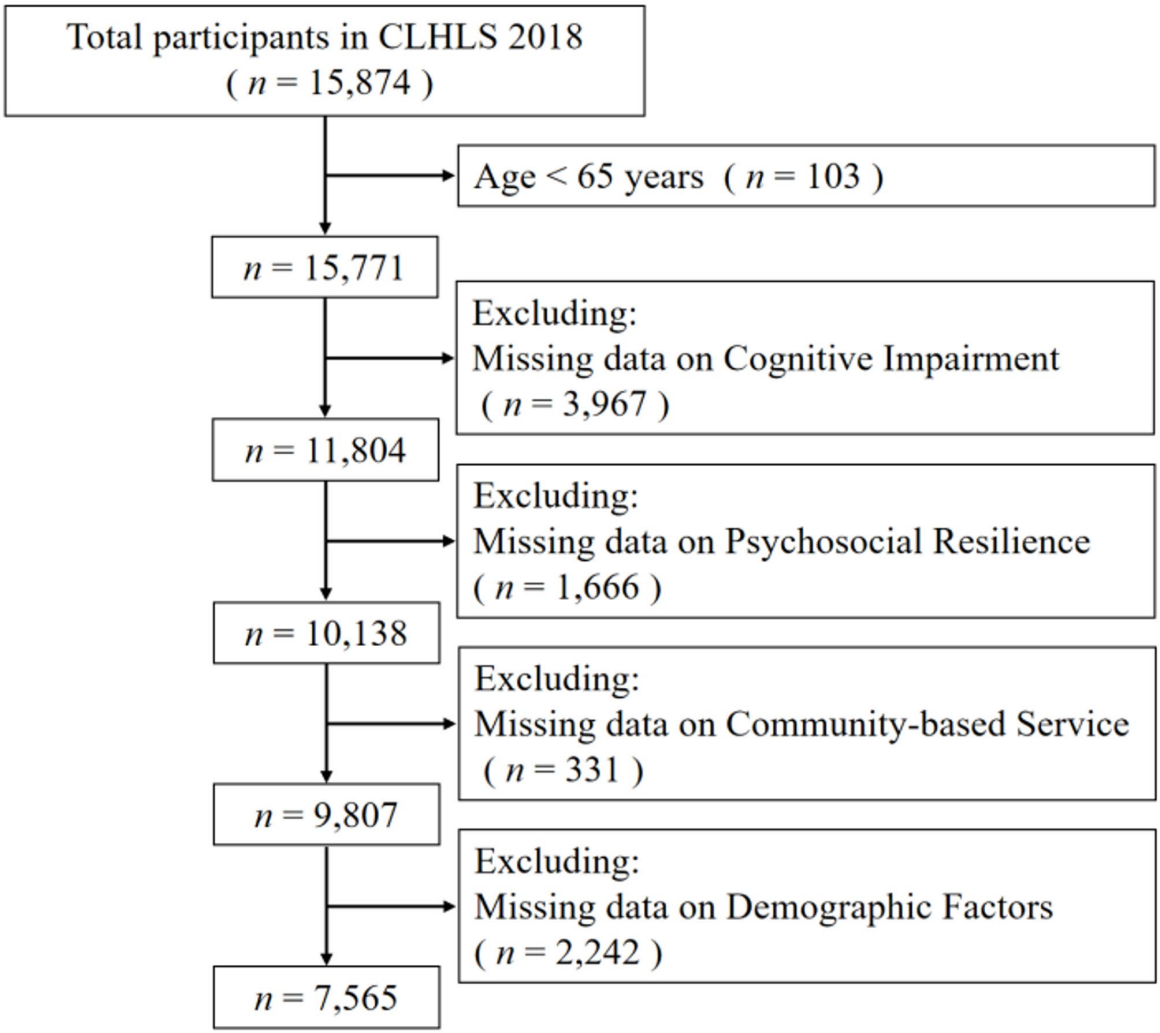
Flowchart showing the selection of the participants enrolled in the CLHLS.

The cross-sectional study formula [ *n* = ((Z_α_/2)^2^pq)/δ^2^ ] was used to establish the appropriate sample size for this investigation. The sample size is denoted by n, the prevalence of CI among older adults is represented by p, while *δ* is the acceptable error determined as 0.1*p*. A two-tailed test Z_α_/2 is set to 1.96, *α* is 0.05, and a two-tailed test is conducted. With the estimated 11.3% prevalence of CI among Chinese older adults, about 3,015 participants were needed for this study to meet its sample size requirements^25^. The inclusion criteria for this study were established as follows: (1) Participants aged 65 years or older who provided written informed consent; (2) Older adults with complete data on CI, PR, CBSs; (3) Senior citizens who provide all information about 18 demographic factors; (4) Absence of severe hearing or visual impairments, major physical comorbidities, or preexisting cognitive disorders. Eventually, 7,565 individuals were included in this study for analysis. Figure 1 below depicts the sample selection flow chart.

### Measures

#### Basic demographic variables

Covariates included eighteen variables that may be significantly related to CI, including age, gender, ethnic group, marital status, residence, living arrangement, body mass index (BMI), drinking, smoking, economic situation, life satisfaction, hypertension, diabetes, heart disease, stroke, physical examination, medical insurance, and social insurance. The detailed information is shown in Supplementary Table S1.

#### Psychological resilience

Seven variables relevant to PR traits are contained in the CLHLS, and the feasibility of the scale has been verified among older adults in other research^26^: (1) Will you be optimistic no matter what difficulties you encounter? (2) Do you enjoy keeping things clean and tidy? (3) Can you decide on your affairs by yourself? (4) Do you feel happy as you did when you were younger? (5) Do you think the older you become, the more useless you are? (6) Will you feel nervous, fearful, or anxious? (7) Will you feel lonely and isolated? The older adults will answer in six options (always = 1, often = 2, sometimes = 3, seldom = 4, never = 5, and unable to answer), and answers with unable to answer will be deleted. Questions 1 to 4 will be assigned the opposite value. These seven characteristics reflect their perseverance, optimism, ability to handle difficult situations, stable relationships, and self-control^27^. Ultimately, the responses to every question will be tallied, and the sum of the scores will fall between 5 and 35. The higher the scores, the more psychologically advanced the older adults are. The Cronbach’s alpha is 0.765, and the Kaiser-Meyer-Olkin (KMO) coefficient is 0.779 in our study.

#### Cognitive impairment

The Mini-Mental State Examination (MMSE) is a widely used cognitive screening tool with 22 questions divided into five categories (general competence, reactivity, attention and numeracy, recollection skills, language, comprehension and self-coordination skills). This comprehensive evaluation, encompassing tasks related to orientation, memory, computation, language, spatial-visual application, and focus, is crucial in determining the precise diagnosis of CI^28,29^. A Chinese version of MMSE is used to analyze the older adults’ cognitive function in our research^30–32^. The detailed description of the scale is shown in Supplementary Table S2.

The total score of the MMSE ranges from 0 to 30, and a higher score demonstrates normal cognitive function. Taking the educational degree into consideration, people with different education levels will be assessed with different standards^33^. If the older adults didn’t experience formal education, scores of 17 or below are considered a CI. If the one to six years of education experience has happened, a score of 20 or less is considered a CI. When they have more than six years of education, they will not be passed as CI unless they score 24 or below^34^. The validity and reliability of the MMSE have been confirmed, with Cronbach’s alpha being 0.878 and the KMO coefficient being 0.842 in this study.

#### Community-based services

Based on previous studies, the community services that older adults have received are used to assess the CBS score^11^. The services included (1) daily care, (2) doctors or sending medicine to home, (3) spiritual comfort, (4) daily shopping, (5) organizing social and recreational activities, (6) providing legal aid (rights protection), (7) provide health care knowledge, (8) handle family and neighborhood disputes, and older adults will answer in two options (Yes = 1, No = 0) (Supplementary Table S3). A total score of about 0 to 8 will be calculated eventually. The higher the score is, the more CBSs they obtain. Our results also confirm that this scale has good validity and reliability among Chinese older adults, with its Cronbach’s alpha being 0.811 and KMO coefficient being 0.856.

### Statistical analysis

The Kolmogorov-Smirnov test is used to determine the normality of continuous variables, and continuous variables that conform to the normal distribution are represented by the mean and standard deviation (M ± SD). The Levene test is further used to verify the assumption of homogeneity of variance between groups. Meanwhile, continuous variables that conform to both normal distribution and homogeneity of variance are further compared for differences in group means using the independent samples t-test and one-way ANOVA. Categorical variables are expressed as percentages and frequencies (*n*(%)). When the expected frequency of a cell in a contingency table is ≥ 5, the Pearson chi-square test is used for examination; when the expected frequency is ≥ 1 but < 5, the continuity-corrected chi-square test is used. Additionally, the Shapiro-Wilk test was used to verify the normality of the total CBS score, the total PR score, the score of five subcategories and the total score of CI. Variables that conform to the normal distribution are further explored for their pairwise correlations using Pearson correlation analysis. SPSS 26.0 is used to complete these statistical analyses.

The SEM was established and the mediation effect was examined using AMOS 26.0 software. Eighteen basic demographic variables potentially associated with the outcome were incorporated into the analytical model. Rigorous statistical control was implemented to minimize their confounding effects on CBSs, PR, and CI, thereby enhancing the robustness and accuracy of the model estimations. The diagonal weighted least squares (WLSMV) method was employed for model estimation, ensuring a more robust handling of the covariance matrix for categorical variables. The verification process employed the Bootstrap 2000 method. Additionally, the study’s test level was *α* = 0.05.

## Results

### Basic characteristics description

A total of 7,565 older adults participated in this research, including 3,541 (46.81%) males and 4,024 (53.19%) females. More than 90% of them are of the Han nationality. In addition, only 1,962 (25.94%) of them live in urban areas, and most of them live in rural areas (40.79%). More than three-quarters of the older adults live with family. A majority of them have good life satisfaction (71.14%), get physical examinations annually (72.27%), and are covered by both medical insurance (58.55%) and social insurance (93.87%). More than 80% of them currently abstain from smoking or drinking, and the prevalence rates of diabetes, heart disease, and stroke all remain below 20% (Table 1).

The mean total score of CBSs was 1.92 ± 2.16, and the prevalence of CI was 11.32% among Chinese older adults. There were statistically significant differences in the total score of CBSs among groups classified by different age, marital status, residence, ethnic group, living arrangement, BMI, smoking, economic situation, life satisfaction, hypertension, diabetes, heart disease, stroke, physical examinations, and medical insurance (*P* < 0.05). Almost all the factors result in a remarkable difference in PR scores except hypertension, diabetes, heart disease, medical insurance, and social insurance(*P* < 0.05). In addition, there is a noteworthy difference in age, gender, marital status, living arrangement, BMI, smoking, drinking, life satisfaction, hypertension, diabetes, stroke, physical examinations, economic situation, medical insurance, and social insurance between older adults with CI. About 8.84% of old males have CI, while about 13.49% of females become ill. Older adults over the age of 80 (17.86%) are seven times more likely to have CI than those under the age of 80 (2.40%). However, those who seldom smoke, drink, or without chronic diseases probably attained a bad CI easily from our results.

**Table 1:** Comparison of community-based services, psychological resilience, and cognitive impairment among different demographic and sociological characteristics (*n* = 7,565).

Variable	*n*(%)	Community -based service (M ± SD)	t/F (*P*)	Psychological resilience (M ± SD)	t/F (*P*)	Cognitive impairment		t/χ^2^ (*P*)
						Yes (*n*%)	No (*n*%)	
Total	7,565(100)	1.92 ± 2.16		26.68 ± 3.99				
Gender								
Male	3,541 (46.81)	1.96 ± 2.17	1.63 (0.103)	27.01 ± 3.93	6.72 (< 0.001)	313 (8.84)	3,228 (91.96)	40.67 (< 0.001)
Female	4,024 (53.19)	1.88 ± 2.16		26.40 ± 4.03		543 (13.49)	3,481 (86.51)	
Age								
< 80	3,203 (42.34)	1.98 ± 2.19	2.04 (0.041)	27.18 ± 3.98	9.34 (< 0.001)	77 (2.40)	3,126 (97.60)	439.59 (< 0.001)
≥ 80	4,362 (57.66)	1.88 ± 2.14		26.32 ± 3.97		779 (17.86)	3,583 (82.14)	
Ethnic group								
Han	7,184 (94.96)	1.97 ± 2.17	10.75 (< 0.001)	26.75 ± 4.01	6.53 (< 0.001)	809 (11.26)	6,375 (88.74)	0.42 (0.519)
Other	381 (5.04)	1.00 ± 1.69		25.51 ± 3.58		47 (12.34)	334 (87.66)	
Marital status								
Married	3,652 (48.27)	1.97 ± 2.16	2.04 (0.041)	27.21 ± 3.89	11.14 (< 0.001)	177 (4.85)	3,475 (95.15)	294.40 (< 0.001)
Other	3,913 (51.73)	1.87 ± 2.17		26.19 ± 4.03		679 (17.35)	3,234 (82.65)	
Residence								
Urban	1,962 (25.94)	2.48 ± 2.51	93.36 (< 0.001)	27.37 ± 4.15	40.61 (< 0.001)	213 (10.86)	6,285 (88.4)	2.21 (0.332)
Town	2,517 (33.27)	1.67 ± 1.88		26.35 ± 3.99		304 (12.08)	1,352 (90.3)	
Rural	3,086 (40.79)	1.77 ± 2.08		26.52 ± 3.84		339 (10.99)	212 (73.9)	
Living arrangement								
Living with family	6,098 (80.61)	1.89 ± 2.11	45.96 (< 0.001)	26.79 ± 3.97	11.23 (< 0.001)	687 (11.27)	5,411 (88.73)	46.84 (< 0.001)
Living alone	1,235 (16.33)	1.80 ± 2.17		26.28 ± 4.09		112 (9.07)	1,123 (90.93)	
Living in institution	232 (3.07)	3.24 ± 2.98		26.06 ± 3.81		57 (24.57)	175 (75.43)	
BMI								
< 18.5 kg/m^2^	1,077 (14.24)	1.65 ± 1.95	11.77 (< 0.001)	25.74 ± 3.87	34.65 (< 0.001)	231 (21.45)	846 (78.55)	148.08 (< 0.001)
18.5–23.9 kg/m^2^	3,915 (51.75)	1.88 ± 2.15		26.61 ± 3.93		431 (11.01)	3,484 (88.99)	
24–27.9 kg/m^2^	1,939 (25.63)	2.08 ± 2.22		27.20 ± 4.04		153 (7.89)	1,786 (92.11)	
≥ 28.0 kg/m^2^	634 (8.38)	2.13 ± 2.32		27.15 ± 4.11		41 (6.47)	593 (93.53)	
Smoking								
No	6,322 (83.57)	1.95 ± 2.18	2.72 (0.006)	26.58 ± 4.02	-5.09 (< 0.001)	762 (12.05)	5,560 (87.95)	20.88 (< 0.001)
Yes	1,234 (16.43)	1.77 ± 2.08		27.19 ± 3.82		94 (7.56)	1,149 (92.44)	
Drinking								
No	6,366 (84.15)	1.90 ± 2.15	-1.78 (0.074)	26.52 ± 3.99	-8.17 (< 0.001)	771 (12.11)	5,595 (87.89)	25.36 (< 0.001)
Yes	1,199 (15.85)	2.02 ± 2.24		27.55 ± 3.91		85 (7.09)	1,114 (92.91)	
Life satisfaction								
Good	5,382 (71.14)	2.03 ± 2.24	23.58 (< 0.001)	27.54 ± 3.69	556.48 (< 0.001)	571 (10.61)	4,811 (89.39)	46.42 (< 0.001)
Fair	1,989 (26.29)	1.66 ± 1.92		24.85 ± 3.79		234 (11.76)	1,755 (88.24)	
Poor	194 (2.56)	1.62 ± 1.97		21.68 ± 4.12		51 (26.29)	143 (73.71)	
Hypertension								
No	4,239 (56.03)	1.84 ± 2.14	3.55 (< 0.001)	26.70 ± 3.99	0.28 (0.778)	544 (12.83)	3,695 (87.17)	22.14 (< 0.001)
Yes	3,326 (43.97)	2.02 ± 2.19		26.67 ± 3.99		312 (9.38)	3,014 (90.62)	
Diabetes								
No	6,719 (88.82)	1.87 ± 2.14	5.21 (< 0.001)	26.73 ± 3.94	2.36 (0.019)	783 (11.65)	5,936 (88.35)	6.85 (0.009)
Yes	846 (11.18)	2.30 ± 2.30		26.35 ± 4.40		73 (8.63)	773 (91.37)	
Heart disease								
No	6,187 (81.78)	1.88 ± 2.14	-3.54 (< 0.001)	26.72 ± 3.93	1.67 (0.096)	717 (11.59)	5,470 (88.41)	2.53 (0.112)
Yes	1,378 (18.22)	2.11 ± 2.25		26.51 ± 4.25		139 (10.09)	1,239 (89.91)	
Stroke								
No	6,720 (88.83)	1.88 ± 2.15	-3.86 (< 0.001)	26.78 ± 3.94	5.75 (< 0.001)	730 (10.86)	5,990 (89.14)	12.26 (< 0.001)
Yes	845 (1.17)	2.20 ± 2.21		25.89 ± 4.32		126 (14.91)	719 (85.09)	
Physical examination								
No	2,098 (27.73)	1.70 ± 2.16	-5.56 (< 0.001)	26.27 ± 4.15	-5.47 (< 0.001)	416 (19.83)	1,682 (80.17)	209.67 (< 0.001)
Yes	5,467 (72.27)	2.00 ± 2.16		26.84 ± 3.92		440 (8.05)	5,027 (91.95)	
Economic situation								
Good	1,575 (20.82)	2.14 ± 2.32	21.29 (< 0.001)	28.11 ± 3.72	242.95 (< 0.001)	136 (8.63)	1,439 (91.37)	26.03 (< 0.001)
Fair	5,284 (69.85)	1.91 ± 2.15		26.58 ± 3.87		608 (11.51)	4,676 (88.49)	
Poor	706 (9.33)	1.51 ± 1.83		24.30 ± 4.25		112 (15.86)	594 (84.14)	
Medical insurance								
No	3,136 (41.45)	1.74 ± 2.10	-6.26 (<0.001)	26.68 ± 3.97	0.01 (0.989)	429 (13.68)	2,707 (86.32)	29.85 (< 0.001)
Yes	4,429 (58.55)	2.05 ± 2.20		26.68 ± 4.01		427 (9.64)	4,002 (90.36)	
Social insurance								
No	464 (6.13)	1.82 ± 2.38	-1.06 (0.291)	26.62 ± 4.02	-0.37 (0.715)	91 (19.61)	373 (80.39)	33.91 (< 0.001)
Yes	7,101 (93.87)	1.93 ± 2.15		26.69 ± 3.99		765 (10.77)	6,336 (89.23)	

*M * mean, *SD* standard deviation, *BMI * body mass index.

### Cognitive impairment in different community-based services

The types of CBSs that are offered are different (Table 2). A majority of the community didn’t provide CBSs, including daily care, spiritual comfort, and daily shopping. Doctors or sending medicine to home, providing health care knowledge, and handling family and neighborhood disputes are more common among all CBSs. About 801 (10.59%) of older adults obtained daily care, while 2,609 (34.49%) of them were provided doctors or sent medicine to home by their community. Spiritual comfort was offered for 1,064 (14.06%) older adults, and 817 (10.80%) of them acquired daily shopping. In addition, the number of older adults who gain social and recreational activities service, legal aid (rights protection), health care knowledge, and family and neighborhood disputes is 1,750 (23.13%), 1,681 (22.22%), 3,255 (43.03%), and 2,968 (33.4%). However, only whether providing a doctor or sending medicine to home (χ^2^ = 7.05, *P* = 0.008), and providing health care knowledge (χ^2^ = 7.89, *P* = 0.005) are associated with older adults’ CI (*P* < 0.05).

**Table 2:** Comparison of cognitive impairment in different kinds of community-based services.

Types (Community-based Service)		*n* (%)	Cognitive Impairment *n* (%)	Normal Cognition *n* (%)	χ^2^ (*P*)
Daily care	Yes	801 (10.59)	705 (88.01)	96 (11.99)	0.40 (0.527)
	No	6,764 (89.41)	6,004 (88.76)	760 (11.24)	
Doctor or send medicine to home	Yes	2,609 (34.49)	2,279 (87.35)	330 (12.65)	7.05 (0.008)
	No	4,956 (65.51)	4,430 (89.39)	526 (10.61)	
Spiritual comfort	Yes	1,064 (14.06)	933 (87.69)	131 (12.31)	1.23 (0.268)
	No	6,501 (85.94)	5,776 (88.85)	725 (11.15)	
Daily shopping	Yes	817 (10.80)	727 (88.98)	90 (11.02)	0.08 (0.775)
	No	6,748 (89.20)	5,982 (88.65)	766 (11.35)	
Social and recreational activities	Yes	1,750 (23.13)	1,574 (89.94)	176 (10.06)	3.59 (0.058)
	No	5,815 (76.87)	5,135 (88.31)	680 (11.69)	
Provide legal aid (rights protection)	Yes	1,681 (22.22)	1,508 (89.71)	173 (10.29)	2.26 (0.133)
	No	5,884 (77.78)	5,201 (88.39)	683 (11.61)	
Provide health care knowledge	Yes	3,255 (43.03)	2,925 (89.86)	330 (10.14)	7.89 (0.005)
	No	4,310 (56.97)	3,784 (87.80)	526 (12.20)	
Handle family and neighborhood disputes	Yes	2,544 (33.63)	2,274 (89.39)	270 (10.61)	1.88 (0.170)
	No	5,021 (66.37)	4,435 (88.33)	586 (11.67)	

Note: χ^2^ *= chi-square test.*

### Correction analysis of community-based services, psychological resilience, and cognitive impairment

The results of the Pearson correlation analysis demonstrated that PR is positively associated with CBSs (*r* = 0.063) and CI (*r* = 0.240). The CBSs also have a positive association between general competence (*r* = 0.051), reactivity (*r* = 0.024), attention and numeracy (*r* = 0.050), recollection skills (*r* = 0.041), language, comprehension, and self-coordination skills (*r* = 0.005), and CI (*r* = 0.050). Other dimensional associations are shown in Table 3.

**Table 3:** Correction analysis of community-based services, psychological resilience and cognitive impairment.

Variable	1	2	3	4	5	6	7	8
1. Community-based Services	1	–	–	–	–	–	–	–
2. Psychological Resilience	0.063**	1	–	–	–	–	–	–
3. General competence	0.051**	0.210**	1	–	–	–	–	–
4. Reactivity	0.024**	0.123**	0.471**	1	–	–	–	–
5. Attention and numeracy	0.050**	0.217**	0.499**	0.473**	1	–	–	–
6. Recollection skills	0.041**	0.117**	0.387**	0.489**	0.498**	1	–	–
7. Language, comprehension and self-coordination skills	0.005**	0.196**	0.506**	0.516**	0.525**	0.432**	1	–
8. Cognitive Impairment	0.050**	0.240**	0.808**	0.698**	0.828**	0.689**	0.751**	1

Note: **P < 0.01; 1 ~ 8 indicate variable numbers.

### Mediating effect of psychological resilience on the relationship between community-based services and cognitive impairment

**Fig. 2:**
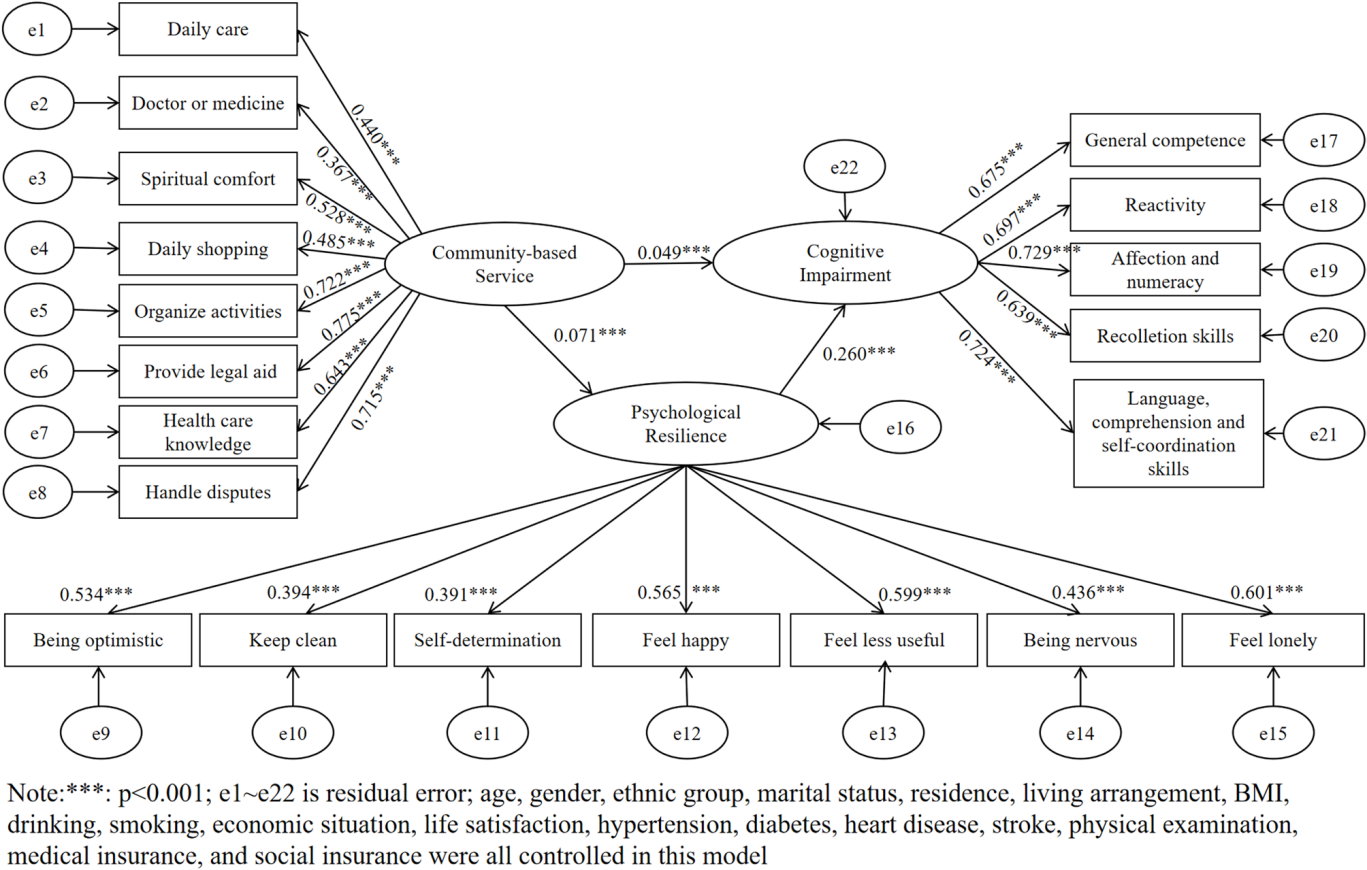
Structural equation model diagram of community-based services, psychological resilience, and cognitive impairment.

Figure 2 presents the results of the mediating effect model. PR had a mediating role in the association with CBSs and CI based on the association between CBSs, PR, and CI. Eighteen variables associated with key variables, including age and gender, were incorporated into the SEM framework, with rigorous control for their potential confounding effects on CBSs, PR, and CI. In the SEM, we constructed using CBSs as the predictor variable (X), PR as the mediating variable (M), and CI as the outcome variable (Y). A good match was found between the model and χ^2^ = 3,426.64, and df = 165. Furthermore, because of the sensitivity to a large sample, the Chi-square value is significant^35^. The standardized root mean square residual (SRMR) = 0.042 (less than 0.05, which we consider a strong model fit), the root mean squared error of approximation (RMSEA) = 0.050 (standardized to less than 0.08), the goodness-of-fit index (GFI) = 0.956, adjusted goodness of fit index (AGFI) = 0.945 (criterion is greater than 0.90), incremental fit index (IFI) = 0.920, comparative fit index (CFI) = 0.920, relative fit index (RFI) = 0.903, normative fit index (NFI) = 0.916, Tucker–Lewis index (TLI) = 0.908 (criterion is greater than 0.90), parsimony fit index (PGFI) = 0.752, parsimony fit index (PCFI) = 0.799, and adjusted normative fit index (PNFI) = 0.795 (criterion is greater than 0.50). The model was deemed acceptable as each significant variable regarding the factor loading on the relevant latent variables was substantial.

According to the findings, CBSs is positively associated with both PR (*β* = 0.071, *P* < 0.001) and CI (*β* = 0.049, *P* < 0.001). Furthermore, PR is positively associated with CI (*β* = 0.260, *P* < 0.001). Y = 0.071X + e1; M = 0.071X + e2; and Y = 0.049X + 0.260 M + e3 were the three models used to forecast the mediating effect (e1, e2, and e3 stand for the regression residuals from the three regressions, respectively).

The bias-corrected Bootstrap 2,000 method was used to test the mediating effect of PR on the relationship between CBSs and CI. The 95% CI for the total, direct, and indirect effect coefficients did not contain 0, indicating that PR partially mediates the relationship between CBSs and CI, with a mediating effect of 0.018. The model *R*^2^ was 52.4% (Table 4).

**Table 4:** The mediating effect of psychological resilience on the relationship between community-based services and cognitive impairment.

Community-based Services → Psychological Resilience → Cognitive Impairment	β	*P*	95%CI		The ratio of total effect
			Lower	Upper	
Total effects	0.067	0.001	0.041	0.094	100%
Direct effects	0.049	0.001	0.023	0.075	73.13%
Indirect effects	0.018	0.002	0.010	0.026	26.87%

Note: *β is the standardized path coefficient; 95% CI is 95% confidence interval*.

## Discussion

Based on the CLHLS database, our study confirmed that CBSs have a positive association with both PR and CI among older adults. Good CBSs are associated with a better CI of older adults by improving their PR. This finding has a certain guiding significance for accelerating the construction of the CBS system and promoting active and healthy aging.

The results indicate a positive correlation between CBSs and CI, which aligns with findings from previous studies^36,37^. However, our results revealed that the effect sizes of CBSs with PR (*r* = 0.063, *P* < 0.01) and CBSs with CI (*r* = 0.063, *P* < 0.01) were relatively small, but still highly significant in the correction analysis. We believe this might be due to the large sample size amplifying this significance. According to the principle of statistical power, when the sample size is large enough, even weak correlations between variables can be statistically detected^38,39^. To further confirm that this significant relationship with a small effect size is not due to bias, we further used Bootstrap 2000 repeated sampling to demonstrate the stability and non-accidental nature of this effect. Additionally, we found that the direct effect (*β* = 0.049) and the mediating effect (*β* = 0.018) of CBSs on CI were relatively small, which might be attributed to the complexity of the etiology of the research subjects, as the occurrence of CI is usually the result of multiple intertwined influencing factors. It is common in sociological and psychological research for *β* coefficients to show small effect sizes (*β* < 0.10), which does not diminish their importance. For instance, a research from Zhang N and other researchers in 2023 explored the mediating effects of loneliness and anxiety between spousal health and the social participation among older adults, finding that spousal health had a significant but small impact on the social participation through the independent mediating effect of loneliness (*β* = 0.020), the independent mediating effect of anxiety (*β* = 0.018), and the chain mediating effect of loneliness and anxiety (*β* = 0.004)^40^. In a study exploring the mediating role of PR in the relationship between CBSs and life satisfaction among older adults, the direct effect and indirect effect of CBSs on life satisfaction were *β* = 0.013 and *β* = 0.004, respectively^41^. From the perspective of public health prevention and intervention, CBS intervention is feasible with relatively low cost and side effects, potentially offering high cost-effectiveness. Secondly, our research population is a nationally representative sample of older adults. According to the results of the seventh national census in 2020, there are currently 264 million older adults aged 60 and above in China^1^. Based on the concept of “population attributable risk” in public health, even if the effect size of a risk factor is small, if the exposed population is large, the overall impact may still be significant. Finally, we must consider the cumulative influence of the effect. For example, long-term exposure to small-effect risk factors may have significant long-term impacts. Or the combined effect of multiple small-effect factors may have a significant impact on health. However, whether this effect size can be further accumulated through continuous intervention requires further exploration through subsequent cohort studies.

In the single-factor analysis, we found that only two specific CBSs—communities offering doctors or sending medicine to home, and those providing healthcare knowledge—had a significant impact on reducing the prevalence of CI. Community with doctors or sending medicine to home ensures that older adults take their medications correctly and on time. For those requiring long-term medication, missed or incorrect doses can lead to poor disease management, which may negatively affect their cognitive function. Regular doctor visits also enable the timely detection of potential health risks, such as abnormal fluctuations in blood pressure or blood sugar levels, which are closely associated with the development of CI. These forms of material assistance and medical support help alleviate persistent anxiety and reduce feelings of insecurity among older adults. Communities that provide healthcare knowledge, through activities such as health lectures and skill training, offer cognitive stimulation to older adults. This stimulation is beneficial for maintaining neuroplasticity, delaying hippocampal atrophy, and further reducing the risk of CI^42^. Additionally, such initiatives strengthen the construction of social support networks and enhance social connectedness among older adults, mitigating risks associated with loneliness or chronic stress, such as activation of the hypothalamic-pituitary-adrenocortical axis, glucocorticoid resistance, and enhanced myelopoiesis^43^. These effects contribute to a lower incidence of cardiovascular-related conditions, such as hypertension and stroke, thereby further improving cognitive function among older adults^44–46^. These findings underscore the importance of targeted CBSs in promoting cognitive health and overall well-being among older adults.

Results further indicated that older adults with high PR scores tend to demonstrate a higher cognitive level. This association may stem from the fact that elevated PR levels reflect richer psychological resources, including optimism, adaptive capacity, and perceived control, which enable effective coping with adversity, trauma, threats, and other adverse life experiences, thereby reducing susceptibility to mental health disorders^47,48^. A concrete manifestation is that older adults with higher PR can promptly mobilize these psychological resources when facing challenges. They are more likely to proactively seek external support for problem-solving rather than resorting to avoidance behaviors. In addition, we found a positive association between CBSs and PR. Daily care, healthcare knowledge dissemination, doctor visits, home delivery of medications, and other CBSs likely enhance the overall health and well-being of older adults. These services allow older adults to experience the warmth and robust social support from the community, which is critical for maintaining normative psychological functioning. In summary, the greater the access to CBSs, the easier it is to meet both the physical and psychological needs of older adults, thereby improving their PR. This highlights the critical role of comprehensive CBSs in fostering PR and promoting mental health among them.

Our study innovatively reveals that PR mediates the relationship between CBSs and CI among older adults. Specifically, CBSs can enhance their PR levels, thereby improving cognitive function and reducing the prevalence of CI. The ABC theory of emotion, proposed by American psychologist Albert Ellis in the 1950s, provides a robust framework for interpreting our findings. The preventive mechanism of CBSs on CI among older adults can be deconstructed into a dynamic interaction system of “Activating Event (A) - Belief (B) - Consequence (C),” where the pathway operates by intervening in external supportive events (A) to reshape individual cognitive beliefs (B), ultimately improving CI (C)^49,50^. As observable real-life events (A), CBSs can directly influence the PR status among older adults in our model, which is in line with the ABC theory’s definition that “A is the objective cause of emotional responses”. The differences in the types of CBSs can form different “A scenarios”, further leading to individualized beliefs (B) and outcomes (C). In our research, it is shown that only “community with doctor or send medicine to home, and providing health care knowledge” has a significant impact on the occurrence of CI, which is consistent with the ABC theory’s mechanism that “the same event can trigger different responses due to different contexts”. Therefore, as an exogenous activating event (A), CBSs provide older adults with essential support and environments, facilitating rational belief restructuring through the enhancement of PR. This process enables older adults to better cope with external challenges. Specifically, CBSs enhance older adults’ sense of social participation, provide health knowledge and psychological support, and help them develop more optimistic self-perceptions and coping mechanisms. For example, through knowledge dissemination, case studies about demonstrations of successful aging, and positive feedback loops, CBSs help correct maladaptive beliefs such as “aging inevitably leads to loss of abilities” (e.g., “I must depend on others”) and replace them with growth mindsets such as “I can manage decline through strategies.” Additionally, various community activities promote the acceptance of aging and the establishment of a sense of meaning in life, mitigating the negative regulatory effects of existential anxiety on the default mode network. The reinforcement of self-efficacy and the reconstruction of meaning systems signify enhanced PR, which further promotes positive emotional and behavioral outcomes (C) through adaptive beliefs (B). These outcomes include increased stress resistance, improved problem-solving abilities, and greater life satisfaction. Enhanced PR enables older adults to better cope with challenges such as cognitive decline, reducing the risk of accelerated CI due to negative emotions and maladaptive coping strategies. In summary, Ellis’s ABC theory provides an operational framework for understanding the intervention efficacy of CBSs on CI among older adults, emphasizing the transformative mechanism from environmental support to cognitive reappraisal. This framework offers significant implications for developing public health policies aimed at enhancing PR among older adults. Therefore, in the context of multi-level elderly care services, it is imperative to improve community health service systems, providing regular and high-quality health services to older adults. Simultaneously, attention should be paid to monitoring changes in the psychological states of older adults, conducting timely assessments and regular follow-ups, and continuously increasing the supply of mental health services. These efforts will advance preventive measures for mental health in older adults, improve cognitive function, and enhance their overall quality of life.

## Limitation

However, there are several limitations to this research. First and foremost, participants with severe hearing or vision impairment, severe physical illness or CI who were unable to complete the questionnaire were excluded from the study. This may lead to an overrepresentation of relatively healthy older adults and affect the estimated prevalence of CI. However, the cognitive function of older adults is directly related to the accuracy of responses. Severe CI patients (such as those in the middle to late stages of Alzheimer’s disease) may have memory loss, language comprehension difficulties or impaired executive function, making it impossible for them to correctly understand the questionnaire questions or recall key information, resulting in data distortion and significantly affecting the accuracy of the results. They are also more likely to have incomplete questionnaires or drop out halfway, leading to an increase in the rate of missing data and further reducing the statistical power of the analysis. Secondly, the use of self-report questionnaires may have introduced recall bias. In addition, due to the constraints of the questionnaire design, our measurement of CBSs is limited to whether they are provided, and there is a lack of relevant information, such as frequency. Nevertheless, our study can also provide a certain reference for the exploration of the relationship between CBSs, PR, and CI among them. It is necessary to point out that although SEM can explore the complex relationship network among variables, due to the cross-sectional design of this study, we are unable to well explain the causal relationship between variables, and can only reveal the statistical correlation. This represents an important direction for future research, which could consider longitudinal studies to better understand the dynamic effects of CBSs on cognitive health in aging populations.

## Conclusion

Our study demonstrates that all hypotheses are validated by the model, indicating that CBSs showed a significant positive association with PR and the cognitive level of older adults. Furthermore, we found that the mediating mechanism of PR is statistically significant. CBSs may enhance PR by providing adequate emotional support to older adults, thereby improving the cognitive level of older adults and reducing the occurrence of CI. Interventions such as doctors or sending medicine to home, including social and recreational activities should be prioritized when improving or promoting community or social health support. And our results showed that approximately 11% of individuals aged 65 and above reported CI. Consequently, older adults should actively adjust their mindset, engage in appropriate physical exercise, reduce unhealthy lifestyle habits, establish regular routines, and enhance pr and cognitive abilities to consciously reduce the risk of cognitive disorders. Additionally, communities and society should collaborate to provide comprehensive physical and mental support to older adults, promoting active and healthy aging. It is the responsibility of the community to provide nursing services, offer psychological counseling services, or psychological knowledge to build a health support system actively. Establishing a culture of care and support for older adults is not only a social responsibility but also an inevitable trend in addressing the needs of an aging society.

## Electronic supplementary material

Below is the link to the electronic supplementary material.

Supplementary Material 1

## Data Availability

The data sets used and analyzed in this study are available from the corresponding author upon request. And all data can be found in this link. 10.18170/DVN/WBO7LK.
