# Supplementary material for: Association between community-based services, psychological resilience, and cognitive impairment among older adults in China
Source: Sci Rep. 2025 Jul 1;15:21210. doi: 10.1038/s41598-025-03491-w (PMC12215793; doi:10.1038/s41598-025-03491-w)
Supplement: Supplementary file 1 — Supplementary Material 1 [file 41598_2025_3491_MOESM1_ESM.docx]

**Association between Community-based Services, Psychological Resilience, and Cognitive Impairment among Older Adults in China**

Suyan Wang^1,2,†^, Xiyu Chen^3,†^, Haoyu Wang^2,†^, Qiwei Tang^3^, Cheng Cheng^1^, Yao Wu^4^, Jinrui Hu^1^, Yishan Duan^1^, Xiaobing Xian^5,6^, Bojiang Chen^1,2,*^

^1^ Frontiers Science Center for Disease-related Molecular Network, West China Hospital, and Key Laboratory of Bio-Resource and Eco-Environment of Ministry of Education, College of Life Sciences, Sichuan University, Chengdu, 610041, Sichuan, China

^2^ Precision Medicine Center, Precision Medicine Key Laboratory of Sichuan Province, West China Hospital, Sichuan University, Chengdu, 610041, Sichuan, China

^3^ The First Clinical College, Chongqing Medical University, Chongqing, 400016, China

^4^ International Medical College, Chongqing Medical University, Chongqing, 400016, China

^5^ The Thirteenth People’s Hospital of Chongqing, Chongqing, 400053, China

^6^ Chongqing Geriatrics Hospital, Chongqing, 400053, China

^†^ These authors contributed equally to this work.

^*^ Corresponding Author: Bojiang Chen

**Email:**

Bojiang Chen: chenbojiang001@163.com

**Supplementary materials**

### Supplementary Table S1 Questionnaire items and classification criteria of covariates

| **Variables** | **Classification** |
| --- | --- |
| Age | 0 = <80, 1 = ≥80 |
| Gender | 0 = Female, 1 = Male |
| Ethnic group | 0 = Han, 1 = Other |
| Marital status | 0 = Married, 1 = Other |
| Residence | 0 = Urban, 1 = Town, 2 = Rural |
| Living arrangement | 0 = Living with family, 1 = Living alone, 2 = Living in institution |
| Body mass index | 0 = <18.5kg/m^2^, 1 = 18.5-23.9kg/m^2^, 2 = 24-27.9kg/m^2^, 3 = ≥28.0kg/m^2^ |
| Smoking | 0 = No, 1 = Yes |
| Drinking | 0 = No, 1 = Yes |
| Life satisfaction | 0 = Good, 1 = Fair, 2 = Poor |
| Hypertension | 0 = No, 1 = Yes |
| Diabetes | 0 = No, 1 = Yes |
| Heart disease | 0 = No, 1 = Yes |
| Stroke | 0 = No, 1 = Yes |
| Physical examination | 0 = No, 1 = Yes |
| Economic situation | 0 = Good, 1 = Fair, 2 = Poor |
| Medical insurance | 0 = No, 1 = Yes |
| Social insurance | 0 = No, 1 = Yes |

### Supplementary Table S2 Questions about the MMSE scale.

| **Classification** | |
| --- | --- |
| General competence  (12 marks) | Question 1: What time is it, morning, noon, afternoon, or evening (correct=1, incorrect or unable to answer=0) |
|  | Question 2: What month is it (correct=1, incorrect or unable to answer=0) |
|  | Question 3: When is the Mid-Autumn Festival (correct=1, incorrect or unable to answer=0) |
|  | Question 4: What season is it (correct=1, incorrect or unable to answer=0) |
|  | Question 5: Name the district or commune you live (correct=1, incorrect or unable to answer=0) |
|  | Question 6: Name the things that can be eaten (1 mark for 1 correct answer, 7 marks for 7 or more answers) |
| Reactivity  (3 marks) | Question 1: Name the “table” correctly (correct=1, incorrect or unable to answer=0) |
|  | Question 2: Name the “apple” correctly (correct=1, incorrect or unable to answer=0) |
|  | Question 3: Name the “clothes” correctly (correct=1, incorrect or unable to answer=0) |
| Attention and numeracy  (6 marks) | Question 1: 20–3=? (correct=1, incorrect or unable to answer=0) |
|  | Question 2: 20–3-3=? (correct=1, incorrect or unable to answer=0) |
|  | Question 3: 20–3-3-3=? (correct=1, incorrect or unable to answer=0) |
|  | Question 4: 20–3–3-3-3-3=? (correct=1, incorrect or unable to answer=0) |
|  | Question 5: 20–3–3-3-3-3-3=? (correct=1, incorrect or unable to answer=0) |
|  | Question 6: Draw the figure on the card (correct=1, incorrect or incomplete=0) |
| Recollection skills (3 marks) | Question: Repeat “table, apple, clothes” as remembered in the “responsiveness section”(1 mark for 1 correct answer; do not count the order of answers) |
| Language, comprehension, and self  coordination skills (6 marks) | Question 1: Name the object the investigator is pointing to as “pen” (correct=1, incorrect or unable to answer=0) |
|  | Question 2: Name the “watch” to which the investigator is referring (correct=1, incorrect or unable to answer=0) |
|  | Question 3: Repeat the assigned sentence from the investigator (correct=1, incorrect or unable to answer=0) |
|  | Question 4: Ask the respondent to hold the paper in their right hand (correct=1, incorrect or unable to complete=0) |
|  | Question 5: Ask the respondent to fold the paper in half (correct=1, incorrect or unable to complete=0) |
|  | Question 6: Ask the respondent to place the paper on the floor (correct=1, incorrect or unable to complete=0) |

### Supplementary Table S3 The measuring tool of community-based service

| **Variables** | **Classification** |
| --- | --- |
| Daily care | 0 = No, 1 = Yes |
| Doctors or sending medicine to home | 0 = No, 1 = Yes |
| Spiritual comfort | 0 = No, 1 = Yes |
| Daily shopping | 0 = No, 1 = Yes |
| Organizing social and recreational activities | 0 = No, 1 = Yes |
| Providing legal aid (rights protection) | 0 = No, 1 = Yes |
| Provide health care knowledge | 0 = No, 1 = Yes |
| Handle family and neighborhood disputes | 0 = No, 1 = Yes |
